# Embolization of left anterior descending artery due to pledget after a redo surgery

**DOI:** 10.1093/jscr/rjae261

**Published:** 2024-04-26

**Authors:** Takeyuki Kanemura, Yoshinori Nakahara, Toshiya Fukushima, Shuhei Kawamoto, Kazuki Morooka, Motoharu Shimozawa

**Affiliations:** Department of Cardiovascular Surgery, IMS Katsushika Heart Center, 3-30-1 Horikiri, Katsushika Ward, Tokyo 124-0006, Japan; Department of Cardiovascular Surgery, IMS Katsushika Heart Center, 3-30-1 Horikiri, Katsushika Ward, Tokyo 124-0006, Japan; Department of Cardiovascular Surgery, IMS Katsushika Heart Center, 3-30-1 Horikiri, Katsushika Ward, Tokyo 124-0006, Japan; Department of Cardiovascular Surgery, IMS Katsushika Heart Center, 3-30-1 Horikiri, Katsushika Ward, Tokyo 124-0006, Japan; Department of Cardiovascular Surgery, IMS Katsushika Heart Center, 3-30-1 Horikiri, Katsushika Ward, Tokyo 124-0006, Japan; Department of Cardiovascular Surgery, IMS Katsushika Heart Center, 3-30-1 Horikiri, Katsushika Ward, Tokyo 124-0006, Japan

**Keywords:** embolization, redo surgery, pledget

## Abstract

A 53-year-old man underwent aortic root replacement for acute aortic dissection. Following this procedure, the patient developed a pseudoaneurysm at the aortic root, necessitating reoperation. The subsequent surgery was performed routinely, allowing the patient to be weaned from mechanical ventilation on the same day. Postoperative electrocardiography revealed ST-segment elevation, suggesting myocardial ischaemia. Coronary angiography identified 90% stenosis in the left anterior descending artery, and computed tomography revealed a high-density mass. These findings suggested an embolus from a previous surgery. A snare catheter was successfully employed to extract the embolic material, which was identified as a pledget used for aortic valve replacement in the initial operation. This case underscores the potential for complications associated with pledgets used in valve surgeries, illustrating the risk of embolization when the valve is subsequently removed.

## Introduction

In cases of repeat surgery after valve or root replacement, pannus can grow not only to cover the valve itself but also the suture line and pledgets used to secure the prosthetic valve [1]. It contributes minimal risk of embolization of pledgets and may be left in place. However, in the case we describe, an embolism in the left anterior descending (LAD) artery occurred postoperatively due to incomplete pledget removal, highlighting the critical importance of thorough management of pledgets during the redo surgical procedures.

## Case report

A 53-year-old male patient underwent aortic root replacement for acute aortic dissection. Subsequently, he developed a pseudoaneurysm at the aortic root, requiring a series of reoperations, including aortic and mitral valve replacement with reconstruction of the intervalvular fibrous body. These surgeries, deemed urgent, were performed without any ST change or hemodynamic instability suggesting coronary ischemia, enabling the patient to be weaned off mechanical ventilation on the same day.

**Figure 1:**
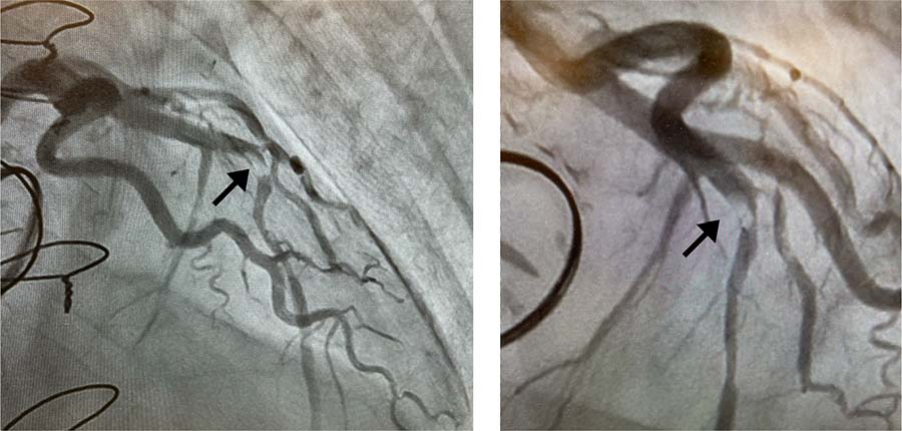
Coronary angiography detected 90% stenosis in the LAD artery; the arrow indicates the filling defect.

**Figure 2:**
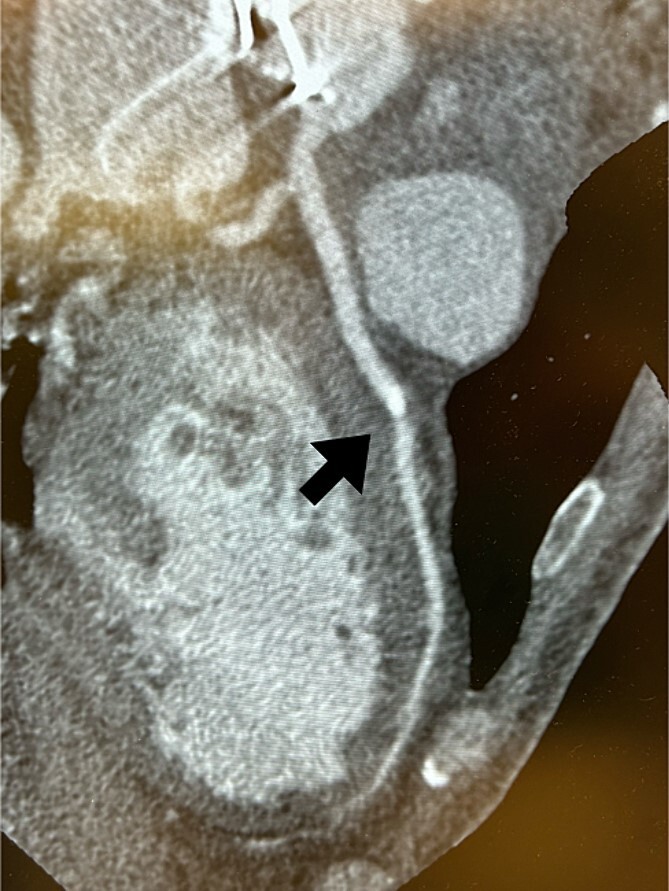
Coronary CT scans revealed a high-density area (arrow) in the LAD artery.

**Figure 3:**
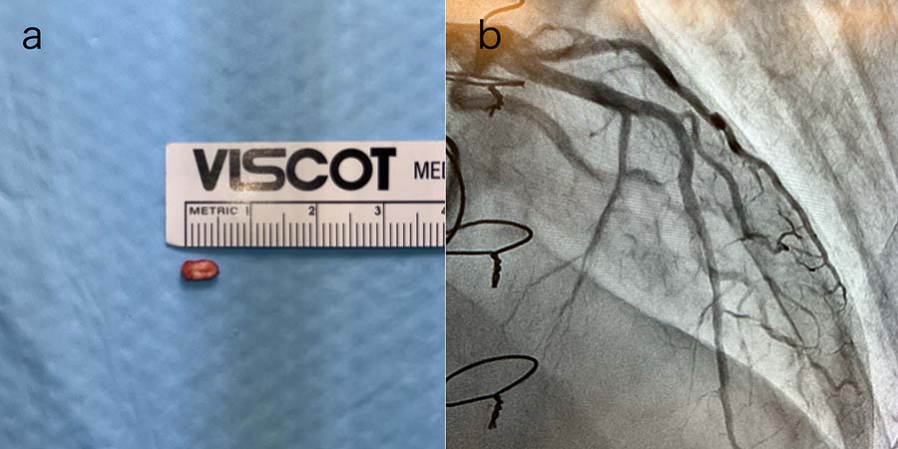
(a) The pledget was removed by transcatheter; (b) TIMI Grade 3 flow in the LAD artery after percutaneous coronary intervention.

Postoperatively, an electrocardiogram revealed anterior ST-segment elevation, indicative of myocardial ischaemia. The patient then developed renal insufficiency. Given his haemodynamic stability, he was initially managed using coronary dilators and heparin. To improve renal function, coronary angiography was performed on the 10th postoperative day, which revealed a filling defect and 90% stenosis in the LAD artery (Fig. 1). Computed tomography (CT) revealed a high-density mass in the LAD artery with a mean CT value of 400 Hounsfield units (HUs) (Fig. 2). These findings indicated embolism, possibly due to a pledget from a previous surgery. A transcatheter approach was employed to remove the embolus, which was successfully extracted using a snare (Fig. 3a). Angiographic assessment revealed that the previously stenotic LAD demonstrated full perfusion, consistent with a thrombolysis in myocardial infarction (TIMI) flow grade of 3 (Fig. 3b). The patient recovered from catheterization without complications.

## Discussion

In this case, the patient experienced an embolism caused by a previous surgical pledget after the operation, which was a redo procedure for an aortic root pseudoaneurysm involving aortic and mitral valve replacement and the reconstruction of the intervalvular fibrous body. It is recommended that pledgets be removed during the redo valve surgery.

First, it was recognized that pledgets remaining from earlier surgeries posed a postoperative embolization risk. We checked the number of pledgets used in the previous surgery at the time of reoperation and tried to remove them all. However, in this case, some of the pledget was not removed as it seemed to be encased in abnormal fibrous tissue. This tissue was identified as pannus because it was associated with the aortic valve and the suture line, and was found beneath the artificial valve [2]. In our search, we found no reports of embolic complications due to a pledget. However, from this case, we infer that it may be impossible to judge whether a pledget is sufficiently covered with a pannus to prevent dispersal. Therefore, all pledgets should be removed if possible.

We found that the pledget stuck in the LAD artery could be safely removed using a catheter. At a heart team conference, a plan to bypass the LAD or remove the foreign body by incising the vessel was discussed. One case report described the successful removal of surgical glue from the left main trunk by directional coronary atherectomy [3]. However, due to repeated open-heart surgeries and the patient’s wishes, a catheter-based treatment plan was decided upon. The embolus was successfully removed by a cardiologist using a snare catheter. Successful transcatheter removal of a catheter migrating into the coronary sinus has been reported as removal of a foreign body from the heart [4]. It is important to note that even emboli in small coronary arteries can be removed using a catheter. This is an important treatment option for cases in which chest reopening is associated with high risk.

In this case, we were able to predict preoperatively that embolization was caused by a pledget. First, the embolus revealed 400 HU on the coronary CT. The causes of embolism after cardiac surgery include blood clots, soft plaques on the arterial wall, and intraoperative use of haemostatic agents. The most frequently reported cases are postoperative embolisms caused by Bio-glu [3, 5–7]. In this case, the 400 HU presented by the embolus was clearly higher than that presented by Bio-Glu. Second, it had a square shape with rounded corners on coronary angiography, suggestive of a manufactured object. These findings suggested that the embolus was an artificial embolism and not due to bio-glue. We inferred that it was a pledget from the previous surgery. CT scan and coronary angiography are useful for estimating the causes of emboli.

## Conclusions

We encountered a complication (LAD embolization) due to an unretrieved pledget during reoperation for a pseudoaneurysm at the aortic root. All pledgets used in the previous valve replacement surgery should be removed during redo cardiac surgery.
